# An Exceedingly Rare Case of Liraglutide-Induced Liver Injury

**DOI:** 10.1155/2021/6306149

**Published:** 2021-08-23

**Authors:** Swetha Parvataneni, Rajarajeshwari Ramachandran, Eric Then, Tyler Grantham, Vinaya Gaduputi

**Affiliations:** ^1^Department of Internal Medicine, Geisinger Lewistown Hospital, Lewiston, Pennsylvania, USA; ^2^Department of Gastroenterology, Brooklyn Hospital Center, Brooklyn, New York, USA; ^3^St. George's University, St. George, Grenada; ^4^Department of Gastroenterology, Blanchard Valley Health System, Findlay, Ohio, USA

## Abstract

Liraglutide is a glucagon-like peptide-1 (GLP-1) receptor agonist used for the treatment of type 2 diabetes mellitus. We are reporting the second case of liraglutide-induced liver injury, with complete resolution of liver injury after discontinuation of the drug.

## 1. Introduction

Diabetes mellitus is a major health burden, affecting about 34.2 million adults in the United States [1, 2]. Several classes of drugs have been developed and approved by the Food and Drug Administration for the treatment of type 2 diabetes mellitus. Glucagon-like peptide-1 (GLP-1) receptor agonists have emerged as a promising treatment for individuals with type 2 diabetes mellitus who have poor glycemic control with oral medications [3]. GLP-1 receptors are present in pancreatic cells, adipose tissue, gastrointestinal tract, central nervous system, and cardiovascular system. Activation of these receptors results in slowed gastric emptying and activation of the satiety center resulting in decreased food intake [4, 5]. These effects underlie the mechanism of GLP-1 agonist in achieving adequate glycemic control and weight loss. Clinical trials demonstrated safety of the drug and its effectiveness in glycemic control when used in combination with other oral antidiabetic medications [6–11]. The most common adverse events reported in these trials were nausea, vomiting, and diarrhea, and rare cases of pancreatitis and thyroid cancer were reported in animal models when exposed to high doses of liraglutide. To our knowledge, this is the second case report of drug-induced liver injury (DILI) directly related to liraglutide, with complete resolution of liver injury upon cessation of the drug.

## 2. Case Report

A 64-year-old woman with hypertension, diabetes mellitus, hyperlipidemia, and history of cholecystectomy presented to the hospital for the evaluation of 4-day history of diffuse abdominal pain. Her home medications were lisinopril, metformin, and liraglutide. She denied alcohol use, illicit drug use, and use of over-the-counter supplements and herbal preparations. On examination, she had epigastric tenderness. She did not exhibit features of liver failure. Her laboratory tests were remarkable for alanine aminotransferase (ALT): 1359 U/L; aspartate aminotransferase (AST): 565 U/L; alkaline phosphatase (ALP): 405 U/L; total bilirubin (TB): 2.9 mg/dL; and INR: 0.9. R factor was consistent with hepatocellular injury. Hepatitis A IgM, hepatitis B surface antigen, hepatitis B surface antibodies, hepatitis B core antibodies and hepatitis C RNA, CMV viral load, and EBV viral load were negative. Immunoglobulin G, antinuclear antibodies, anti-smooth muscle antibodies, antimitochondrial antibodies, anti-liver-kidney microsomal antibodies, ferritin, iron saturation, alpha-1 antitrypsin, ceruloplasmin, and 24-hour urinary copper were normal. Abdominal ultrasound demonstrated fatty changes of the liver, without evidence of biliary obstruction. Her liver tests were normal 6 months prior to presentation, when the patient was started on liraglutide. Due to concern for DILI, liraglutide was discontinued and N-acetylcysteine was administered. On the third day of hospitalization, the patient's symptoms resolved, and her liver tests demonstrated gradual improvement. On the day of discharge, her liver tests were ALT: 335 U/L; AST: 58 U/L; ALP: 286 U/L; and TB: 1.3 mg/dL. 2 months after discharge, her liver tests normalized.

## 3. Discussion

The liver is the major site for first-pass metabolism of medications. DILI is the most common cause of acute liver failure in the United States [12, 13]. The incidence of idiosyncratic DILI has risen because of increased emergence of new medications with the growing chronic medical problems.

Liraglutide is evolving as a promising treatment option for patients with type 2 diabetes mellitus. It is a recombinant human GLP-1 analog with 97% amino acid sequence homologous with human GLP-1 [14]. Mechanism of liver injury due to liraglutide use is unknown, and the medication label includes elevation of liver enzymes based on postmarketing experiences. Approximately 8% of patients exposed to liraglutide will develop antiliraglutide antibodies; however, in the study by Buse et al., the antiliraglutide antibodies were found to have no effect on the safety of medication use [14].

In our literature search, we identified a case of liraglutide-induced autoimmune hepatitis reported by Kern et al; however, the patient's condition did not improve after stopping liraglutide, and the patient eventually needed long-term corticosteroid therapy [15]. Lack of improvement after discontinuation of the drug in this case suggests autoimmune hepatitis independent of the drug use or liraglutide triggered underlying liver condition [16]. Maor et al. reported the first care of liraglutide-induced hepatotoxicity in a 53-year-old woman taking the medication as part of her weight loss program, with complete resolution of liver injury after discontinuation of the medication [17].

Our patient had a hepatocellular pattern of liver injury in the setting of recent initiation of liraglutide. Liver biopsies are not required to make the diagnosis of DILI. Based on the temporal relationship between the use of liraglutide and development of liver injury, we suspected liraglutide-induced liver injury. Our patient was advised to discontinue liraglutide leading to normalization of liver tests and this further supported our diagnosis of liraglutide-induced liver injury.

## Abbreviations

GLP-1: Glucagon-like peptide-1
DILI: Drug-induced liver injury.
